# Healthy Selfishness and Pathological Altruism: Measuring Two Paradoxical Forms of Selfishness

**DOI:** 10.3389/fpsyg.2020.01006

**Published:** 2020-05-21

**Authors:** Scott Barry Kaufman, Emanuel Jauk

**Affiliations:** ^1^Department of Psychology, Columbia University, New York, NY, United States; ^2^Clinical Psychology and Behavioral Neuroscience, Technische Universität Dresden, Dresden, Germany; ^3^Department of Psychology, University of Graz, Graz, Austria

**Keywords:** selfishness, vulnerable narcissism, pathological altruism, depression, well-being

## Abstract

Selfishness is often regarded as an undesirable or even immoral characteristic, whereas altruism is typically considered universally desirable and virtuous. However, human history as well as the works of humanistic and psychodynamic psychologists point to a more complex picture: not all selfishness is necessarily bad, and not all altruism is necessarily good. Based on these writings, we introduce new scales for the assessment of individual differences in two paradoxical forms of selfishness that have lacked measurement in the field – healthy selfishness (HS) and pathological altruism (PA). In two studies (*N*_1_ = 370, *N*_2_ = 891), we constructed and validated the HS and PA scales. The scales showed good internal consistency and a clear two-dimensional structure across both studies. HS was related to higher levels of psychological well-being and adaptive psychological functioning as well as a genuine prosocial orientation. PA was associated with maladaptive psychological outcomes, vulnerable narcissism, and selfish motivations for helping others. These results underpin the paradoxical nature of both constructs. We discuss the implications for future research, including clinical implications.

“*Any pleasure that does no harm to other people is to be valued.*”– Russell (1930)

“*What we value so much, the altruistic ‘good’ side of human nature, can also have a dark side. Altruism can be the back door to hell.*” – Oakley et al. (2012)

## Introduction

We tend to think of altruism as unselfish and beneficial, with minimal tradeoffs, and selfishness as generally bad and glutinous, negatively impacting on others. Reality points to a much more complex story. There are many examples across human history of the unintended negative consequences of altruism on the self and others, despite the best intentions. Oakley et al. (2012) refer to this as “pathological altruism” and note that “some of human history’s most horrific episodes have risen from people’s well-meaning altruistic tendencies” (p. 3). They use the example of Oliver Wendell Holmes, a well-respected American Supreme Court justice, whose well-intentioned rhetoric supported eugenic forced sterilization. On the flip side, Maslow (1943/1996) noted that “healthy selfishness”— a healthy respect for one’s own health, growth, happiness, joy, and freedom— can have a *positive* impact both on the self and on others.

While there has been some theory and indirect evidence (e.g., vignettes and historical examples) of these paradoxical forms of selfishness, there is a dearth of empirical evidence systematically investigating individual differences in healthy selfishness and pathological altruism. We believe this is due, in large part, to the lack of reliable and valid scales to capture these constructs. In the current study, we present new scales of both healthy selfishness and pathological altruism and distinguish them from related constructs in the field. By doing so, we hope to add more nuance to both the concepts of “selfishness” and “altruism” and offer new research tools for researchers to further investigate individual differences in these understudied topics, which have important societal and clinical implications.

## Healthy Selfishness

In his 1939 essay “Selfishness and Self-Love,” Erich Fromm opened by declaring that “Modern culture is pervaded by a taboo on selfishness. It teaches that to be selfish is sinful and that to love others is virtuous.” In his essay, Fromm argues that this cultural taboo has had the unfortunate consequence of making people feel guilty to show themselves *healthy* self-love, which he defines as the passionate affirmation and respect for one’s own happiness, growth, and freedom.

Fromm argues that the form of selfishness that society decries— an interest *only* in oneself and the inability to give with pleasure and respect the dignity and integrity of others— is actually the *opposite* of self-love. To Fromm, love is an attitude that is indiscriminate of whether it is directed outward or inward. In contrast, Fromm argued that selfishness is a kind of greediness: “Like all greediness, it contains an instability, as a consequence of which there is never any real satisfaction. Greed is a bottomless pit which exhausts the person in an endless effort to satisfy the need without ever reaching satisfaction” (Fromm, 1939).

Inspired by Fromm’s essay, Maslow (1943/1996) wrote an essay in which he argued for the need to clearly distinguish “healthy selfishness” from unhealthy selfishness, as well as the importance of distinguishing healthy and unhealthy motivations for one’s seemingly selfish behavior.

Defining selfishness as any behavior that brings any pleasure or benefit to the individual, Maslow argued that: “For our part, we must not prejudge the case. We must not assume that selfish or unselfish behavior is either good or bad until we actually determine where the truth exists. It may be that at certain times, selfish behavior is good, and at other times, it is bad. It also may be that unselfish behavior is sometimes good and at other times bad.” Maslow goes on to note that “a good deal of what appears to be unselfish behavior may come out of forces that are psychopathological and that originates in selfish motivation” (p. 110).

Calling for the need for a new vocabulary that incorporates the notion of healthy selfishness, Maslow noted that in the process of psychotherapy it is sometimes necessary to teach people at certain times to engage in a “healthfully selfish manner”— to have a healthy respect for one’s self that stems from abundance and need gratification— that “comes out of inner riches rather than inner poverty” (p. 110).

A recent meta-analysis of the literature on communion supports these early ideas. Le et al. (2018) found that communally motivated people who care for the welfare of others and their close relationship partners experience greater relationship well-being. However, personal well-being was maximized *only to the extent that people were not self-neglecting in their communal care*. Therefore, while the health and relationship benefits of promoting the well-being of others has been well-documented (Crocker and Canevello, 2008, 2018), the role of healthy selfishness in contributing to well-being and relationships may have been neglected in the literature.

## Pathological Altruism

According to Crocker and Canevello (2008, 2018), humans evolved two systems: an “egosystem” that is motivated by a desire for positive impressions from others, and an “ecosystem,” which is motivated by the promotion of the well-being of others by fostering their thriving and avoiding harm to them. Critically, Crocker and Canevello (2018) argue that sometimes people who are motivated by the egosystem can act in prosocial ways “not because they genuinely care about others’ well-being and want to be constructive and supportive, but instead as a strategy to manage others’ impressions” (p. 52).

While intriguing, this idea has not been tested extensively in the psychological literature. The study of altruism has mostly focused on the positive benefits of altruism, and how humans are wired to care for the welfare and suffering of others (Keltner, 2009; Vaillant, 2009; Ricard, 2013). However, as Bachner-Melman and Oakley (2016) note, “Western societies have become so focused on its benefits that its flip side has been virtually ignored” (p. 92). Examples of pathological altruism range widely from genocide, suicide martyrdom, to codependency (Oakley et al., 2012).

Early psychoanalytical writings focused on the dark side of altruism, and the selfish motives that can underlie it. Anna Freud introduced the concept of *altruistic surrender* to describe a situation in which a person who is unable to achieve direct gratification of instinctual wishes can achieve vicarious gratification through a proxy (Freud, 1946). Anna Freud saw a prime illustration of altruistic surrender in the drama character of Cyrano de Bergerac; a poet of exceptional talent, but unblessed in physical appearance. Cyrano is in love with his beautiful cousin Roxane, but afraid of her rejection, and thus surrenders his own desires to another man, helping him to win Roxanes’ heart by writing love letters.

While Anna Freud thought of altruism as mostly synonymous with altruistic surrender, later work in psychoanalytic theory acknowledged the healthy functions of altruism. Vaillant (1977) argued that altruism is one of the healthiest defense mechanisms and found that it predicted lifelong positive relationships and personal fulfillment. Nevertheless, Vaillant’s clinical examples of altruism were similar to Anna Freud’s description of altruistic surrender, a compromise of need deprivation resulting in finding a proxy in whom to satisfy one’s own impulses and fantasies (Seelig and Rosof, 2001).

More recent psychoanalytic theory has more carefully and explicitly distinguished between healthy altruism and pathological altruism (Seelig and Rosof, 2001). Presenting a more comprehensive system of classification, Seelig and Rosof (2001) argued that mature and *healthy altruism*—“the ability to experience sustained and relatively conflict-free pleasure from contributing to the welfare of others” can be distinguished from *pathological altruism*, “a need to sacrifice oneself for the benefit of others.” They argue that the individual with healthy altruism can gratify their needs directly, regulate their affect, and also enjoy enhancing the good of others.

A big boon to the understanding of the science of pathological altruism came with the publication of the edited volume “Pathological Altruism” in 2012 (Oakley et al., 2012). In this book and a subsequent article (Oakley, 2013), the authors make a call to subject altruism to more systematic scientific inquiry. Oakley et al. (2012) brought a wide variety of perspectives to bear on pathological altruism, from sociology to evolutionary biology to clinical psychology. As Oakley (2013) put it, “it is time for dispassionate exploration of how altruism and empathy themselves can inadvertently bias our efforts to create truly co-operative modern, complex societies.” (p. 2)

In a later book chapter, Bachner-Melman and Oakley (2016) defined pathological altruism as “the willingness of a person to irrationally place another’s perceived needs above his or her own in a way that causes self-harm” (p. 92). They argued that major motivations in healthy altruism are openness to new experiences and a desire for personal growth, whereas the major motivation for individuals with pathological altruism is to please others, gain approval, and avoid criticism and rejection. They gave examples of individuals with eating disorders, codependency in relationships, political extremism, and even cancer caregiving (“those whose care for cancer patients reaches self-harming extremes turn out, interestingly, to be unable to comfortably receive care themselves”, p. 93).

Bachner-Melman and Oakley (2016) also linked pathological altruism to narcissism, arguing that “narcissism and altruism may in fact represent two sides of the same coin” (p. 99). In particular, they linked pathological altruism to “hypervigilant narcissism” (more commonly referred to in the modern scientific literature as vulnerable narcissism; see Kaufman et al., 2018). According to the researchers, at the core of the inner world of those with pathological altruism is a deep sense of shame related to their secret wish to display themselves and their needs in a grandiose manner. Stemming from a lack of a sense of self, attention is continually directed toward others, reading, anticipating, or attempting to guess others’ needs and giving them priority over their own real needs.

Developmentally, Bachner-Melman and Oakley (2016) drew on the work of Heinz Kohut, who argued that healthy development requires having one’s needs appreciated or “mirrored” in the eyes of significant others. Kohut argued that if such mirroring is not met early in life, an exaggerated need for responsiveness from others develops, and a healthy sense of self-esteem is less likely to be established (Kohut, 1971). Such children may grow ashamed of their desire to be seen and valued, and ashamed of their dependence on others for support. They may attempt to lighten that burden and shame by being as undemanding as possible and a brittle facade of self-sufficiency sets in as a result. Underneath the facade, however, often lies anger, frustration, and resentment at having to sacrifice so much and receive so little in return.

It’s an interesting and open question whether a reliable and valid scale of pathological altruism would show strong correlations with vulnerable narcissism as well as with the early developmental experience of having to substitute one’s own needs for the needs of others.

## Current Studies

To construct new scales of pathological altruism and healthy selfishness, we mined descriptions of these concepts from Fromm (1939), Maslow (1943/1996), Oakley et al. (2012), Oakley (2013), and Bachner-Melman and Oakley (2016). Based on the theoretical arguments of these writers, we could make some predictions.

In terms of healthy selfishness, we expected healthy selfishness to show moderately negative correlations with pathological altruism, but to not simply be the opposite of pathological altruism. In particular, we predicted that healthy selfishness would be more strongly tied to sociality, positive relationships, and other dimensions of well-being than pathological altruism. To highlight the paradoxical nature of healthy selfishness, we also predicted that healthy selfishness would be positively correlated with prosocial traits (e.g., the light triad; see Kaufman et al., 2019) and prosocial motivations (a genuine satisfaction for helping others), and to be distinct from other forms of unmitigated agency (overdominance and control over others; Helgeson and Fritz, 1999) such as measures of narcissism and other forms of unhealthy selfishness.

We expected pathological altruism to be fundamentally motivated by selfish concerns, but for those selfish concerns to be primarily about a fear or rejection and fear of losing emotional intimacy stemming from low self-esteem rather than the more grandiose narcissistic motives for exploitation, power, and control over others. In particular, we predicted that pathological altruism would be correlated with low self-esteem and high vulnerable narcissism as well as higher levels of communally oriented aspects of narcissism including self-sacrificing self-enhancement (Pincus et al., 2009) and communal narcissism (Gebauer et al., 2012), but show weaker correlations with grandiose narcissism overall and more exploitative selfish motivations (which we refer to as “unhealthy selfishness”). Due to the pathological nature of the construct, we also expected pathological altruism to be more strongly tied to negative outcomes such as depression than the abundance of well-being.

Finally, we expected both healthy selfishness and pathological altruism to show ties to unmitigated communion (Helgeson, 1994; Fritz and Helgeson, 1998; Helgeson and Fritz, 1999). Fritz and Helgeson (1998) demonstrated that unmitigated communion—over-involvement in the problems and suffering of others— is distinct from communion in terms of a negative view of the self, turning to others for self-evaluative information, and psychological distress. They found that those scoring higher in unmitigated communion tended to show higher levels of psychological distress due to their reliance on others for self-esteem and validation, which led to overinvolvement with others and a neglect of the self. They also found that those scoring high in unmitigated communion scored higher in intrusive thoughts about the problems of others, pointing to a compulsive nature of unmitigated communion.

Considering that the Unmitigated Communion Scale includes a mix of items relating to *self-neglect* (“I *always* place the need of others above my own”) and *an overconcern with the problems of others* (“I worry about how other people get along without me when I am not there”; Fritz and Helgeson, 1998, p. 140), we expected that pathological altruism would show a strong positive correlation with unmitigated communion and healthy selfishness would show a moderate *negative* correlation with unmitigated communion. However, we expected that pathological altruism and healthy selfishness would predict important outcomes above and beyond unmitigated communion.

One criticism of the Unmitigated Communion Scale is that it does not adequately differentiate between different underlying motives for self-sacrifice (Bassett and Aubé, 2013). Indeed, Helgeson and Fritz (1999) admit this when they write that “some unmitigated communion individuals’ involvement in other people’s problems may be motivated by a need to have control over relationships, as relationships can be a source of self-esteem” (p. 155). Bassett and Aubé (2013) attempted to distinguish between self-sacrifice that is motivated by concern for others versus self-sacrifice that is motivated by concern for the self. They gave participants the Unmitigated Communion Scale and then asked them to rate their underlying motive for their answer to each item. They showed that it is possible to score high in unmitigated communion for different reasons: it’s possible to score high in unmitigated communion for *self-oriented reasons* (being motivated by a desire to feel affirmed or valued by others) or score high in unmitigated communion for *other-oriented reasons* (being motivated by a genuine care and concern for the well-being of others). They found that other-oriented motives for unmitigated communion predicted higher levels of secure attachment whereas self-oriented motives for unmitigated communion were related to a preoccupied attachment style (which consists of negative views of the self and a positive view of others) and shame.

Their study highlighted the importance of considering the motivation underlying behavior rather than just the behavior itself. In the current study, we expected that pathological altruism would be more clearly related to selfish motivations for helping others as well as maladaptive psychological adjustment (e.g., fear, depression) than unmitigated communion, and that healthy selfishness would be more clearly tied to prosocial motivations for helping others as well as healthy sociality and overall psychological adjustment (including positive relationships and life satisfaction) than unmitigated communion.

## Study 1: Scale Development

The aim of Study 1 was the item selection and initial validation of the healthy selfishness (HS) and pathological altruism (PA) scales, especially concerning measures that are closely conceptually related. We selected items on the basis of conceptual considerations, psychometric characteristic, and exploratory factor analysis. Validity was assessed with respect to conceptually related constructs, particularly unmitigated communion, and indicators of adaptive and maladaptive psychological adjustment (low levels of life satisfaction and high levels of depression).

## Methods

### Participants and Procedure

An online sample of *N* = 370 (171 female) English-speaking participants was acquired via Amazon’s Mechanical Turk platform. Data from participants who failed our attention checks were removed from further analysis. The large majority of participants (76%) reported residing in the United States during the time of testing. The mean age was 37.67 years (*SD* = 12.29). Among the full sample, 70.3 % self-identified as Caucasian, 8.1% as Hispanic, and 4.9% as African American; the rest did not disclose their ethnical origin. Concerning educational status, one participant (0.30%) did not complete high school, 27.80% of participants completed high school, 71.80% had a bachelor’s degree or higher. Participants gave written informed consent to the study, took part on a voluntary basis, and received monetary compensation. The study was approved by the ethics committee of the University of Pennsylvania.

### Measures

The main aim of Study 1 was item selection for the HS and PA scales. For this, we administered an initial pool of 16 candidate items designated to assess HS, and candidate 19 items for the assessment of PA. The items were constructed by the first author on the basis of conceptual considerations outlined in the introduction section. Participants answered the items on a five-point scale ranging from “Disagree strongly” to “Agree strongly.”

Additionally, participants completed measures of *unmitigated communion* (original 8-item unmitigated communion scale; Helgeson, 1993), the *light triad* of personality (12-item scale assessing Kantianism, Humanism, and Faith in Humanity which can be scored as a general light triad factor; Kaufman et al., 2019), *life satisfaction* (5-item The Satisfaction with Life Scale; Diener et al., 1985), and *depression* (20-item Center for Epidemiological Studies Depression scale, CES-D (Radloff, 1977). We used those latter two scales as proxies for adaptive and maladaptive psychological adjustment, paralleling the more comprehensive analyses in Study 2 (see below).

Additionally, we also administered seven newly devised items measuring underlying motivations for helping others (e.g., “A major reason why I help people is to gain approval from them”) and two newly devised items assessing possible childhood antecedents that might differentiate between HS and PA (e.g., “As a child, I was often encouraged by my family to substitute my own needs for their own”). The percentage of missing data in this pilot study was low, not exceeding 1% on any single variable.

## Results

### Item Selection

Almost all items from the initial HS and PA item pool displayed psychometrically satisfactory difficulty between *p* = 0.20 and 0.80 (0.21 < *p* < 0.74; see Table 1); we excluded one PA item which did not meet this criterion (*p* = 0.19; “my helping sometimes causes others harm”).

**TABLE 1:** Principal component analysis and item-level statistics of the Healthy Selfishness and Pathological Altruism scales in Study 1.

**No.**	**Item**	**Component 1 (HS)**	**Component 2 (PA)**	***p***	***r*_i–s_**
	*Healthy Selfishness (HS)*				
1	I have healthy boundaries.	**0.71**	−0.07	0.72	0.66
2	I have a lot of self-care.	**0.82**	0.13	0.61	0.67
3	I have a healthy dose of self-respect, and don’t let people take advantage of me.	**0.70**	−0.13	0.69	0.69
4	I balance my own needs with the needs of others.	**0.74**	0.10	0.68	0.61
5	I advocate for my own needs.	**0.61**	−0.06	0.64	0.56
6	I have a healthy form of selfishness (e.g., meditation, eating healthy, exercising, etc.) that doesn’t hurt others, but brings me greater happiness.	**0.71**	0.05	0.69	0.62
7	Even though I give a lot to others, I know when to recharge.	**0.71**	0.07	0.68	0.59
8	I give myself permission to enjoy myself, even if it doesn’t necessarily help others.	**0.53**	−0.06	0.72	0.51
9	I take good care of myself.	**0.79**	0.06	0.69	0.67
10	I prioritize my own personal projects over the demands of others.	**0.50**	−0.18	0.59	0.52
	*Pathological Altruism (PA)*				
1	I tend to sacrifice my own needs and interests so that I can devote myself to helping and serving others.	0.11	**0.75**	0.49	0.59
2	I am a total pushover when it comes to requests to help others.	−0.17	**0.56**	0.44	0.57
3	I often feel a compulsion to help others, as though I can’t help myself.	0.05	**0.75**	0.41	0.64
4	I am willing to place another’s needs above my own in a way that may cause self-harm.	−0.03	**0.73**	0.39	0.65
5	I am constantly trying to read, anticipate, or guess others’ needs so that I can give them exactly what they want.	0.08	**0.72**	0.49	0.59
6	I have little time to myself because I am too busy helping everyone.	0.07	**0.82**	0.35	0.70
7	I often suffer from “empathy burnout”– helping others leaves me feeling exhausted.	−0.16	**0.63**	0.39	0.65
8	I need to be needed.	0.13	**0.70**	0.50	0.53
9	I often feel run down due to the demands of others.	−0.09	**0.69**	0.42	0.67
10	I often feel unappreciated for the work I do to help others.	−0.21	**0.48**	0.45	0.53

**N* = 370. Bold numbers indicate theoretically expected loadings.*

The HS items displayed high initial item-scale correlations (0.43 < *r*_i–s_ < 0.68), with the exception of one item (*r* = 0.37; “I notice when there are problems in my relationships and I try to fix the situation”), which we excluded. We further excluded five items which were conceptually less clear and distinctive than the others (e.g., “I ask for help when I’m feeling low” or “I look after myself so that I can better help others”). The remaining item-scale correlations were 0.51 < *r*_i–s_ < 0.69 (see Table 1). The internal consistency of the 10-item scale was α = 0.88. The scale skewness indicated a long left tail (*z* = −5.34, *p* < 0.001), which means that a greater number of people show higher than lower HS in the present scale. The scale kurtosis conformed to a normal distribution (*z* = 1.72, *p* = 0.08).

Among the PA items, item-scale correlations were also high (0.49 < *r*_i–s_ < 0.71). Again, we excluded items that were conceptually less clearly related to PA, albeit correlated (e.g., “I try very hard to look attractive, even if I have to sacrifice my own health to do so”). After exclusion of eight items, the final 10-item scale (paralleling the length of the HS scale) displayed item-scale correlations between 0.53 < *r*_i–s_ < 0.70 (see Table 1), similar to the HS scale. The overall internal consistency was α = 0.88, just like the HS scale. The scale skewness conformed to normality (*z* = −1.97, *p* = 0.05), and scale kurtosis indicated a platykurtic distribution (*z* = −3.45, *p* < 0.001).

### Factor Structure

We conducted a principal components analysis to validate the intended factor number of the HS and PA scales. Velicer’s (1976) original and revised (Velicer et al., 2000) MAP test indicated a two-factor solution. The first principal component accounted for 35.82% of variance, the second for 13.30%. The component correlation after Promax rotation was *r* = −0.45, indicating that the components underlying HS and PA are moderately anticorrelated. The components clearly distinguished HS and PA items, with intended loadings ≥ 0.48 and cross-loadings ≤ 0.21 (see Table 1). Thus, the analysis clearly confirms the intended two-factor structure of the HS and PA scales (a further confirmatory analysis is provided in Study 2).

### External Validity

Table 2 displays the correlations between HS and PA, demographical variables, and other variables assessed in this study. HS displayed a slight positive association with participants’ age, PA displayed a negative association. None of the two forms of paradoxical selfishness were associated with participants’ sex.

**TABLE 2:** Descriptive statistics and correlations of Study 1 variables.

	***M* (*SD*)**	**2**	**3**	**4**	**5**	**6**	**7**	**8**
**Demographic variables**								
Age (1)	37.67 (12.29)	0.20	0.12	−0.24	−0.14	0.26	0.05	−0.30
Sex (2)	0.46 (0.50)		0.02	0.00	0.01	0.10	0.02	−0.02
**Paradoxical selfishness**								
Healthy Selfishness (3)	3.69 (0.74)			−0.48	−0.42	0.29	0.47	−0.49
Pathological Altruism (4)	2.74 (0.90)				0.71	−0.05	−0.14	0.54
**Validity measures**								
Unmitigated Communion (5)	3.05 (0.80)					0.13	−0.11	0.44
Light Triad (6)	3.89 (0.57)						0.30	−0.32
Life Satisfaction (7)	3.37 (1.13)							−0.53
Depression (8)	1.99 (0.62)							

**N* = 370. Correlations greater *r* = 0.10, *r* = 0.13, and *r* = 0.17 are significant at *p* = 0.05, *p* = 0.01, and *p* = 0.001, respectively. Sex: Men are coded 0, Women are coded 1.*

HS and PA were moderately negatively correlated, paralleling the principal components correlation reported above. We expected PA to be positively correlated with unmitigated communion, whereas HS should be negatively related. HS and PA should further display opposing relationships with the light triad, life satisfaction, and depression. As expected, PA displayed a high positive correlation with unmitigated communion, while HS displayed a negative relationship. HS was further positively correlated with the light triad, highly positively correlated with life satisfaction, and highly negatively related to depression. PA displayed a slight negative association with life satisfaction, and a rather strong positive association with depression.

We had a-priori interest in the predictive power of the HS and PA scales on measures of adaptive and maladaptive psychological adjustment (in Study 1: life satisfaction and depression). To investigate the incremental validity of the HS and PA scales beyond unmitigated communion, we conducted hierarchical multiple regression models for the prediction of life satisfaction as an indicator of adaptive psychological functioning, and depression as an indicator of maladaptive functioning. We used unmitigated communion and HS/PA as predictor variables and investigated the effects of unmitigated communion alone (step 1: equals zero-order correlations from Table 2), steps 2a, b: unmitigated communion and HS/PA, and step 3: unmitigated communion, HS, and PA).

Table 3 displays the results. In step 1, unmitigated communion displayed a slight negative relationship with life satisfaction and a strong positive association with depression. When we entered HS in step 2a, we found HS to be a strong predictor of life satisfaction and depression, and the effects of unmitigated communion were still significant. When entering PA in step 2b, we observed no significant effects on life satisfaction. PA was the only significant predictor of depression. This pattern was reflected in step 3, which shows that when all variables are considered simultaneously, HS is the only predictor of life satisfaction, and PA is the strongest predictor of depression (though HS is also a strong predictor, although in the opposite direction).

**TABLE 3:** Hierarchical multiple regression models for the prediction of psychological functioning indicators in Study 1.

	**Adaptive functioning: Life satisfaction**	**Maladaptive functioning: Depression**
**Step 1**		
Unmitigated Communion	**−0.11**	**0.44**
	*R*^2^_adj_ = 0.01	*R*^2^_adj_ = 0.19
**Step 2a**		
Unmitigated Communion	**0.11**	**0.28**
Healthy Selfishness	**0.52**	**−0.37**
	*R*^2^_adj_ = 0.23	*R*^2^_adj_ = 0.25
**Step 2b**		
Unmitigated Communion	−0.02	0.11
Pathological Altruism	−0.13	**0.46**
	*R*^2^_adj_ = 0.02	*R*^2^_adj_ = 0.29
**Step 3**		
Unmitigated Communion	0.08	0.06
Healthy Selfishness	**0.53**	**−0.29**
Pathological Altruism	0.06	**0.35**
	*R*^2^_adj_ = 0.23	*R*^2^_adj_ = 0.36

**N* = 891. Standardized β coefficients are reported. Coefficients in bold are significant at *p* < 0.05.*

These results demonstrate first evidence for the incremental validity of (HS and) PA above and beyond unmitigated communion, which is important as these are conceptually and empirically related. While these results yield first evidence for convergent and discriminant validity, Study 2 will consider a larger set of external validity measures, more finely differentiating the motives associated with HS and PA.

## Study 2: Scale Validation

The aim of Study 2 was to validate the structure of the newly devised HS and PA scales using confirmatory factor analysis as well as external validity measures in a large sample. To this end, we included a manifold of conceptually related variables to test convergent and discriminant validity. Findings from Study 1 were replicated using more fine-grained measures of the respective constructs. Moving beyond Study 1, we also included an interpersonal circumplex measure to assess the relations of HS and PA with agentic and communal orientations and included a broader array of criterion measures for adaptive psychological adjustment (a multidimensional scale of well-being in addition to life satisfaction) and maladaptive adjustment (a multidimensional scale of fears in addition to depression).

### Participants and Procedure

We acquired an online sample of *N* = 891 (472 female, 2 non gender-identified) participants via Amazon’s Mechanical Turk. Data from participants who failed our attention checks were removed from further analysis. The large majority of participants (89%) resided in the United States during the time of testing. The mean age was 37.12 (*SD* = 11.30) years; 0.80% (seven participants) did not complete high school, 42.30% completed high school, 56.90% had a bachelor’s degree or higher. The research reported here is part of a larger project on personality; part of the data were previously published and the study procedure was described in greater detail (Jauk and Kaufman, 2018). The study was carried out in accordance with the relevant guidelines and regulations. The protocol was approved by the Ethics committee of the University of Pennsylvania. All subjects gave written informed consent in accordance with the Declaration of Helsinki. Participants received monetary compensation.

### Measures

We used the same pool of HS and PA items as in Study 1. Analyses are based on the item selection of Study 1 resulting in 10 items per scale (see Table 1). As in Study 1, we assessed *life satisfaction* (The Satisfaction with Life Scale; Diener et al., 1985), a *modified scale of unmitigated communion* that distinguishes self- and other-oriented unmitigated communion (18-item Two-Dimensional Unmitigated Communion Scale, TUCS; Bassett and Aubé, 2013), and *depression* (CES-D; Radloff, 1977). Additionally, the validation study encompassed a brief measure of the *Big Five* (Ten-Item Personality Inventory, TIPI; Gosling et al., 2003), a measure of *self-esteem* (16-item Self-Liking/Self-Competence Scale-Revised Version SLCS-R; Tafarodi and Swann, 2001), *pathological selfishness* (Selfishness Questionnaire, SQ; Raine and Uh, 2018), the *Light Triad* (Light Triad Scale; Kaufman et al., 2019), *grandiose and vulnerable narcissism* (Five Factor Narcissism Inventory Short Form FFNI-SF; Sherman et al., 2015), the *Self-Sacrificing Self-Enhancement* subscale of the Pathological Narcissism Inventory (PNI; Pincus et al., 2009), *communal narcissism* (Gebauer et al., 2012), the *core motives of achievement, power, affiliation, and intimacy* (Unified Motive Scales UMS; Schönbrodt and Gerstenberg, 2012), *authentic and hubristic pride* (Tracy and Robins, 2007), *psychological well-being* (42-item version of Ryff’s Psychological Well-Being Scale; see for instance Abbott et al., 2006), and *fear* (fear of failure, rejection, losing control, losing emotional contact, and losing reputation from the Unified Motive Scales UMS, which can be aggregated to a composite index; Schönbrodt and Gerstenberg, 2012). We assessed the *interpersonal circumplex* scales using the International Personality Item Pool–Interpersonal Circumplex (IPIP-IC; Markey and Markey, 2009). Lastly, as in Study 1, we administered the same set of items assessing motivation for helping others and childhood antecedents of HS and PA.

### Data-Analytical Strategy

We first re-assessed the factor structure of the HS and PA scales using confirmatory factor analysis (CFA). We then examined correlations to validity measures and to the Interpersonal Circumplex. As the sample is large and almost all correlations are significant at conventional levels, we focus on effect sizes rather than significance levels in the interpretation of results. Next, we investigated the validity of the HS and PA scales on measures of adaptive psychological adjustment (psychological well-being, life satisfaction) and maladaptive adjustment. To this end, we tested the incremental validity of the HS and PA scales above and beyond the Big Five and conceptually related constructs, namely unhealthy selfishness, unmitigated communion (self- and other-directed), and communal narcissism, on the criterion variables psychological well-being, life satisfaction, fear, and depression. These criterion measures tap into the positive and negative poles of general psychological functioning.

We expected that that HS scale would be more predictive of positive psychological adjustment (well-being, life satisfaction), whereas the PA scale would be indicative of psychological maladjustment (fear, depression). Lastly, we investigated the relations between motivation for helping others and the childhood antecedents of HS and PA across both studies.

## Results

### Confirmatory Factor Analyses

**FIGURE 1:**
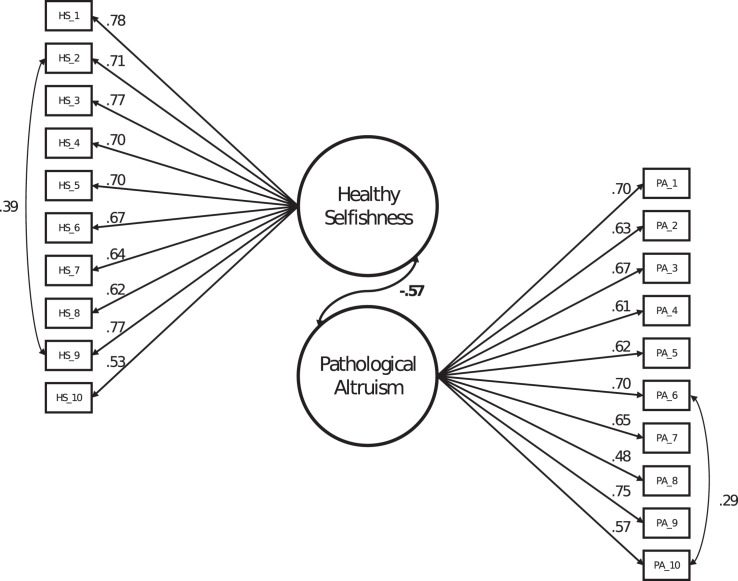
Confirmatory factor analysis model of the Healthy Selfishness and Pathological Altruism scales. Error variables are not displayed.

We conducted CFAs separately for the HS and PA scales and jointly for both scales. Since the results for the single models and the joint model were very similar, we only present the joint CFA of both scales here. The model converged to an admissible solution and displayed acceptable fit to the data (χ^2^(167) = 850.38, *p* < 0.001; RMSEA = 0.07; CFI = 0.82; SRMR = 0.05). While the χ^2^ test was significant, which might be due to its high sensitivity in large samples, the other indices can be deemed acceptable^1^. As Figure 1 shows, factor loadings were high and consistent. The explained variance in the single indicators was significant for each item (*p* < 0.001). We specified one residual correlation per scale to account for unique variance between the items. Importantly, there were no substantial cross-loadings of items or residual correlations between scales (i.e., specifying such effects would not have improved model fit substantially). This confirms the EFA results of Study 1. The latent correlation between the HS and PA factors was *r* = −0.57, also conforming to the result of Study 1.

### Descriptive Statistics and Intercorrelations

**FIGURE 2:**
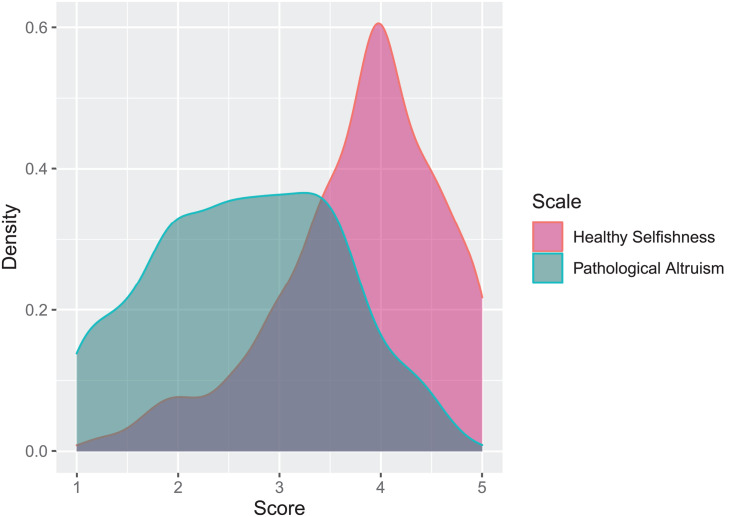
Density distributions of the Healthy Selfishness and Pathological Altruism scales in Study 2. *N* = 891.

Figure 2 displays the distributions of the HS and PA scales. As in study 1, the HS scale displayed a long left tail, indicating that the majority of participants displayed higher rather than lower HS. The PA scale, also as in Study 1, displayed a platykurtic distribution. Table 4 displays the descriptive statistics and correlations between the HS and PA scales with demographic variables, Big Five personality dimensions, conceptually related personality constructs, and indicators of adaptive and less adaptive psychological functioning. The means and *SD*s of the HS and PA scales were almost identical to Study 1. Also, the correlations to age and sex, unmitigated communion (overall), life satisfaction, and depression were highly similar to those obtained in Study 1. Among the Big Five personality traits, HS was positively associated with extraversion and agreeableness, whereas PA was, albeit to a lesser extent, associated with neuroticism and disagreeableness. Both showed weak associations with conscientiousness in opposing directions.

**TABLE 4:** Descriptive statistics and correlations of Study 2 variables.

	***M* (*SD*)**	**2**	**3**	**4**	**5**	**6**	**7**	**8**	**9**	**10**	**11**	**12**	**13**	**14**	**15**	**16**	**17**	**18**	**19**	**20**	**21**	**22**	**23**	**24**	**25**	**26**	**27**	**28**	**29**	**30**	**31**
Demographic variables																															
Age (1)	37.12 (11.30)	0.13	0.09	–0.23	–0.12	0.08	0.08	0.12	–0.06	0.12	0.12	0.09	–0.25	–0.11	–0.16	0.01	0.15	–0.35	–0.30	–0.21	–0.19	0.00	–0.14	–0.15	0.00	–0.01	–0.24	0.05	–0.03	–0.14	–0.16
Sex (2)	0.53 (0.50)		–0.08	0.09	0.04	–0.06	0.05	0.00	0.02	–0.10	–0.10	–0.08	–0.27	0.14	0.08	0.17	0.18	–0.27	–0.37	0.08	–0.08	–0.05	–0.24	–0.15	0.15	–0.13	–0.26	0.03	0.03	0.16	–0.03
Paradoxical Selfishness																															
Healthy Selfishness (3)	3.75 (0.80)			–0.51	–0.06	0.28	0.04	0.23	–0.10	0.68	0.69	0.54	0.08	–0.42	–0.38	–0.23	0.21	0.01	0.23	–0.42	0.21	0.39	0.26	0.32	0.19	0.57	0.02	0.57	0.40	–0.35	–0.50
Pathological Altruism (4)	2.69 (0.90)				0.18	–0.06	–0.05	–0.14	0.16	–0.43	–0.45	–0.31	0.12	0.61	0.62	0.51	0.04	0.34	0.13	0.53	0.26	–0.03	0.03	0.07	0.15	–0.23	0.19	–0.21	–0.11	0.49	0.46
Big Five																															
Neuroticism (5)	3.20 (0.59)					–0.14	–0.06	–0.01	0.23	–0.08	–0.09	–0.05	0.03	0.25	0.23	0.21	0.10	0.15	0.09	0.18	0.11	0.07	0.01	0.00	0.15	–0.01	0.11	0.06	0.06	0.22	0.12
Extraversion (6)	2.69 (1.14)						0.10	0.26	–0.13	0.37	0.37	0.31	0.01	–0.21	–0.14	–0.01	0.26	0.12	0.27	–0.29	0.29	0.29	0.38	0.64	0.24	0.41	0.06	0.38	0.29	–0.23	–0.28
Openness (7)	3.73 (0.99)							0.03	0.03	0.06	0.03	0.12	–0.16	–0.04	–0.10	0.06	0.16	–0.09	–0.09	–0.06	–0.03	0.25	–0.01	0.03	0.03	0.02	–0.15	0.02	–0.07	–0.07	–0.03
Agreeableness (8)	3.58 (1.03)								–0.09	0.30	0.31	0.22	–0.36	–0.03	–0.06	0.16	0.61	–0.32	–0.16	–0.48	0.21	0.19	–0.06	0.33	0.29	0.28	–0.26	0.36	0.28	–0.20	–0.31
Conscientiousness (9)	3.28 (0.64)									–0.19	–0.19	–0.16	0.09	0.17	0.15	0.13	0.02	0.15	0.08	0.22	–0.01	0.01	–0.03	–0.07	0.06	–0.18	0.12	–0.10	–0.10	0.21	0.21
Self-Esteem (10)	3.43 (0.79)										0.96	0.87	–0.06	–0.46	–0.43	–0.23	0.22	–0.04	0.26	–0.58	0.33	0.50	0.34	0.36	0.24	0.78	–0.04	0.71	0.56	–0.53	–0.63
Self-Worth (11)	3.51 (1.09)											0.71	–0.06	–0.46	–0.43	–0.24	0.22	–0.08	0.23	–0.60	0.28	0.41	0.29	0.35	0.23	0.76	–0.04	0.71	0.55	–0.53	–0.65
Self-Competence (12)	3.34 (0.60)												–0.06	–0.35	–0.35	–0.18	0.18	0.04	0.27	–0.43	0.34	0.56	0.36	0.30	0.22	0.66	–0.03	0.59	0.46	–0.42	–0.49
Pathological Selfishness (13)	0.65 (0.54)													–0.05	0.09	–0.18	–0.47	0.66	0.62	0.36	0.11	0.01	0.39	0.07	–0.13	0.05	0.52	–0.09	–0.08	0.11	0.23
Unmitigated Communion (14)	3.19 (0.85)														0.88	0.81	0.19	0.12	–0.13	0.48	0.14	–0.10	–0.19	–0.01	0.19	–0.27	0.04	–0.19	–0.10	0.61	0.34
UC Self (15)	2.95 (0.91)															0.74	0.12	0.26	0.02	0.53	0.21	–0.11	–0.07	0.11	0.20	–0.21	0.13	–0.16	–0.06	0.65	0.34
UC Other (16)	3.37 (0.88)																0.42	0.01	–0.15	0.25	0.20	0.07	–0.12	0.18	0.35	–0.10	–0.07	0.02	0.05	0.46	0.16
Light Triad (17)	3.94 (0.64)																	–0.33	–0.25	–0.33	0.22	0.34	–0.12	0.32	0.45	0.26	–0.30	0.42	0.36	0.00	–0.27
Narcissism (18)	2.60 (0.53)																		0.90	0.59	0.47	0.20	0.62	0.28	0.05	0.14	0.58	–0.03	–0.02	0.27	0.34
Grandiose Narcissism (19)	2.46 (0.69)																			0.21	0.54	0.32	0.71	0.40	0.05	0.38	0.57	0.18	0.16	–0.06	0.13
Vulnerable Narcissism (20)	2.84 (0.78)																				0.02	–0.17	0.05	–0.18	–0.03	–0.42	0.24	–0.43	–0.35	0.69	0.57
Communal Narcissism (21)	2.84 (0.87)																					0.33	0.41	0.51	0.32	0.44	0.22	0.35	0.30	0.03	–0.02
Motives																															
Achievement (22)	3.88 (0.75)																						0.46	0.36	0.33	0.52	0.00	0.44	0.31	–0.16	–0.24
Power (23)	2.65 (0.96)																							0.42	0.15	0.43	0.36	0.23	0.18	–0.06	–0.08
Affiliation (24)	2.87 (0.99)																								0.40	0.48	0.15	0.46	0.39	–0.06	–0.23
Intimacy (25)	3.76 (0.81)																									0.34	–0.13	0.49	0.43	0.19	–0.22
Pride																															
Authentic (26)	3.18 (1.02)																										0.13	0.79	0.69	–0.37	–0.53
Hubristic (27)	1.43 (0.67)																											–0.01	0.03	0.08	0.30
Well-Being (28)	6.96 (1.81)																												0.76	–0.28	–0.64
Life Satisfaction (29)	3.37 (1.15)																													–0.20	–0.53
Fear (30)	3.18 (0.87)																														0.40
Depression (31)	1.91 (0.64)																														

**N* = 891 (except for sex, where *N* = 889). Correlations greater *r* = 0.07, *r* = 0.09, and *r* = 0.11 are significant at *p* = 0.05, *p* = 0.01, and *p* = 0.001, respectively.*

Among the further effects displayed in Table 4, it is interesting to note that HS was substantially positively related to self-esteem (particularly the self-liking facet) whereas PA was *negatively* related to both facets of self-esteem (self-liking and self-competence). Neither HS nor PA were markedly associated with pathological selfishness, suggesting that HS and PA are both independent from an exploitative form of selfishness.

Also as expected, PA was strongly related to unmitigated communion (somewhat stronger with self-oriented unmitigated communion), whereas HS was negatively related to both self-oriented and other-oriented motivations underlying unmitigated communion. To further disentangle these effects, we conducted complemental regression analyses using self- and other-oriented unmitigated communion as predictors of HS and PA. These show that when both motivations underlying unmitigated communion are considered simultaneously, there was a strong negative effect of self-oriented unmitigated communion on HS (β = −0.45, *p* < 0.001), whereas other-oriented unmitigated communion had only a weak effect (β = 0.10, *p* = 0.03). A similar picture emerged for PA, where self-related unmitigated communion was a strong positive predictor (β = 0.55, *p* < 0.001), whereas other-oriented unmitigated communion was not (β = 0.10, *p* = 0.011). The predictive validity of PA and unmitigated communion will be evaluated below.

As in Study 1, HS was correlated with the Light Triad Scale, whereas PA was not. HS was not related to overall narcissism and only weakly correlated with grandiose narcissism, which provides evidence for its conceptual distinctiveness, and, as expected, was negatively correlated with vulnerable narcissism. On the contrary, PA was moderately related to overall narcissism, which was mainly due to the strong correlation with vulnerable narcissism. This is in line with the correlations of PA with neuroticism and disagreeableness (antagonism) observed among the Big Five. However, as we did not expect the correlation between PA and vulnerable narcissism to be so high, we conducted an exploratory analysis between PA and the 15 FFNI subscales. The highest correlations emerged between PA and the subscales *Need for Admiration* (*r* = 0.53, *p* < 0.001), *Shame* (*r* = 0.47, *p* < 0.001), and *Entitlement* (*r* = 0.36, *p* < 0.001). Of note, PA was weakly negatively correlated with a *Lack of Empathy* (r = −0.10, *p* < 0.001). PA was also substantially correlated with the Self-Sacrificing Self-Enhancement Scale (*r* = 0.45, *p* < 0.001) of the PNI (Pincus et al., 2009). Further, HS and PA were both significantly related to communal narcissism (*r*_*HS, communal*_ = 0.21, *p* < 0.001; *r*_*PA, communal*_ = 0.29, *p* < 0.001). Taken together, the pattern of correlations shows that PA taps more into vulnerable narcissism and communal-oriented aspects of narcissism than overall grandiose narcissism, while HS is negatively related to vulnerable narcissism and only slightly positively related to more grandiose forms of narcissism.

Among the core motives, HS was moderately positively associated with achievement, power, and affiliation, whereas PA displayed no pronounced associations with the core motives. Paralleling the finding of an independence of HS from narcissism, HS was strongly associated with authentic pride, but not with hubristic pride. In contrast (and also in line with the narcissism findings), PA was negatively related to authentic pride and even displayed a small positive association with hubristic pride.

Concerning indicators of psychological functioning, as expected, HS was positively related to adaptive functioning in terms of psychological well-being and self-esteem, whereas PA was negatively related to those indicators. PA was, instead, positively associated with indicators of maladaptive functioning in terms of fear and depression. Complemental facet-level analyses for fear yielded largely homogeneous correlations with HS and PA. When we controlled for the shared variance among the different fears using multiple regression models, HS displayed a negative relationship with fear of rejection (β = −0.39, *p* < 0.001), and, to a lesser extent, failure (β = −0.17, *p* < 0.001), and losing control (β = −0.10, *p* = 0.02), whereas it was positively associated with fear of losing reputation (β = 0.20, *p* < 0.001). PA displayed positive associations with fear of losing control (β = 0.21, *p* < 0.001), losing emotional contact (β = 0.20, *p* < 0.001), and rejection (β = 0.15, *p* < 0.001). Incremental validity of the HS and PA scales on measures of adaptive and maladaptive functioning beyond other personality constructs will be tested below.

### Interpersonal Circumplex

**FIGURE 3:**
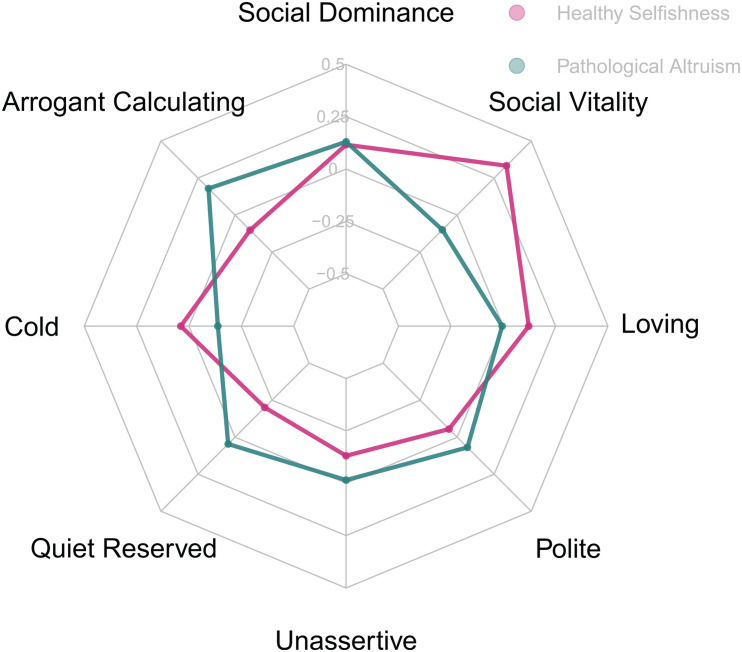
*N* = 891. Correlations of Healthy Selfishness and Pathological Altruism to the Interpersonal Circumplex scales. Axes range from *r* = –0.5 to *r* = 0.5. Correlations greater *r* = 0.07, *r* = 0.09, and *r* = 0.11 are significant at *p* = 0.05, *p* = 0.01, and *p* = 0.001, respectively.

Figure 3 displays the associations between HS and PA and the scales of the interpersonal circumplex. The HS scale displayed the strongest associations with social vitality (*r* = 0.33), and, negatively, quietness and reservedness (*r* = −0.20). HS was thus associated with a friendly assertive interpersonal style. PA was less clearly associated with a particular interpersonal style. Consistent with the paradoxical nature of PA, we observed the strongest positive associations with arrogant/calculating (*r* = 0.18) and social dominance (*r* = 0.13), and the strongest negative association with cold-heartedness (*r* = −0.14). However, note that all of these associations were rather weak. Taken together, it can be concluded that HS is associated with a friendly assertive interpersonal style, whereas PA was less clearly linked to the interpersonal circumplex.

### Incremental Validity Analyses

We tested the explanatory power of the HS and PA scales in separate regression models predicting indicators of adaptive psychological functioning (well-being, life satisfaction) and less adaptive adjustment (fear, depression) above and beyond the broad Big Five traits and personality traits which are conceptually related to HS and PA (unhealthy selfishness, unmitigated communion, communal narcissism). We split unmitigated communion into self- and other-directed aspects as different associations can be expected based on previous findings (Bassett and Aubé, 2013). We set up separate models for HS and PA as we did not have a-priori interest in controlling the two constructs for their respective counterparts, they were moderately anticorrelated, and including them in a model with many predictors might evoke complex suppression effects.

Table 5 displays the hierarchical multiple regression models. For every criterion variable, we first entered the Big Five in step 1, followed by unhealthy selfishness, self-/other-oriented unmitigated communion, and communal narcissism in step 2, and finally HS/PA in step 3a/b, respectively. We will focus on the results in step 3 here, as we were interested in the predictive power of the HS and PA scales above and beyond a complete set of theoretically related constructs. Steps 1 and 2 are displayed for a complete picture of the relative importance of the single predictors.

**TABLE 5:** Hierarchical multiple regression models for the prediction of psychological functioning indicators in Study 2.

	**Adaptive functioning**		**Maladaptive functioning**	
	**Well-being**	**Life Satisfaction**	**Fear**	**Depression**
**Step 1**				
Neuroticism	**0.11**	**0.11**	**0.16**	0.05
Extraversion	**0.31**	**0.25**	−**0.15**	−**0.19**
Openness	−0.01	−**0.09**	−0.05	0.00
Agreeableness	**0.28**	**0.21**	−**0.14**	−**0.25**
Conscientiousness	−**0.07**	−**0.08**	**0.14**	**0.15**
	*R*^2^_adj_ = 0.23	*R*^2^_adj_ = 0.15	*R*^2^_adj_ = 0.13	*R*^2^_adj_ = 0.17
**Step 2**				
Neuroticism	**0.11**	**0.10**	0.04	−0.01
Extraversion	**0.21**	**0.18**	−**0.09**	−**0.18**
Openness	−0.02	−**0.10**	0.00	0.05
Agreeableness	**0.21**	**0.14**	−**0.11**	−**0.19**
Conscientiousness	−0.05	−**0.07**	**0.08**	**0.11**
Unhealthy Selfishness	0.01	−0.04	0.04	**0.13**
Unmitigated Communion Self	−**0.30**	−**0.15**	**0.59**	**0.30**
Unmitigated Communion Other	**0.14**	0.08	0.05	−0.02
Communal Narcissism	**0.27**	**0.22**	−**0.06**	0.01
	*R*^2^_adj_ = 0.31	*R*^2^_adj_ = 0.19	*R*^2^_adj_ = 0.47	*R*^2^_adj_ = 0.25
**Step 3a**				
Neuroticism	**0.10**	**0.09**	0.04	0.01
Extraversion	**0.16**	**0.15**	−**0.08**	−**0.13**
Openness	−0.03	−**0.11**	0.00	**0.06**
Agreeableness	**0.12**	**0.07**	−**0.09**	−**0.11**
Conscientiousness	−0.04	−0.05	**0.08**	**0.10**
Unhealthy Selfishness	−**0.09**	−**0.12**	0.06	**0.22**
Unmitigated Communion Self	−0.08	0.01	**0.56**	**0.09**
Unmitigated Communion Other	**0.10**	0.05	0.05	0.02
Communal Narcissism	**0.17**	**0.14**	−0.05	**0.10**
Healthy Selfishness	**0.46**	**0.34**	−**0.07**	−**0.43**
	*R*^2^_adj_ = 0.46	*R*^2^_adj_ = 0.27	*R*^2^_adj_ = 0.48	*R*^2^_adj_ = 0.38
**Step 3b**				
Neuroticism	**0.11**	**0.10**	0.04	−0.01
Extraversion	**0.22**	**0.19**	−**0.09**	−**0.18**
Openness	−0.02	−**0.10**	0.00	0.05
Agreeableness	**0.17**	**0.11**	−**0.09**	−**0.13**
Conscientiousness	−0.04	−0.06	**0.08**	**0.09**
Unhealthy Selfishness	0.01	−0.05	0.05	**0.14**
Unmitigated Communion Self	−**0.19**	−0.08	**0.53**	**0.12**
Unmitigated Communion Other	**0.18**	**0.11**	0.03	−0.07
Communal Narcissism	**0.31**	**0.24**	−**0.08**	−0.05
Pathological Altruism	−**0.24**	−**0.16**	**0.12**	**0.39**
	*R*^2^_adj_ = 0.34	*R*^2^_adj_ = 0.20	*R*^2^_adj_ = 0.48	*R*^2^_adj_ = 0.34

**N* = 891. Standardized β coefficients are reported. Coefficients in bold are significant at *p* < 0.05. *R*^2^ is significant in all models.*

HS was a strong predictor of well-being and life satisfaction and had greater explanatory power than any other variable included in the models. Also, HS was the strongest negative predictor of depression. HS had, however, only low predictive power for fear. Instead, the strongest predictor of fear was a self-oriented motivation for unmitigated communion. Taken together, HS was a strong predictor of adaptive psychological functioning beyond the Big Five and conceptually related personality constructs.

PA also significantly negatively predicted adaptive functioning, but the effects were weaker than for HS. Instead, PA was the best predictor of depression among all of the variables. Importantly, PA displayed a stronger effect on depression than a self-oriented motive for unmitigated communion when simultaneously considered in a model (despite these variables being strongly related; see Table 4). PA was also predictive of fear, but, here, self-oriented unmitigated communion displayed the stronger effect. Taken together, we conclude that PA is more indicative of maladaptive than adaptive adjustment, particularly depression. To this end, PA demonstrated higher predictive power than conceptually related personality constructs.

### Motivations and Childhood Antecedents

As an additional exploratory analysis, we investigated the relations of HS and PA to a set of newly devised items on motivation for helping others, overly nurturant, other-harming helping behavior, and possible childhood antecedents that might discern HS from PA.

Table 6 shows the item-level correlations across both studies. HS was consistently *negatively* associated with items that assess helping others for self-oriented motives (items 1−4; e.g., “A major reason why I help people is to gain approval from them”), whereas PA was strongly *positively* associated with these items. To this end, approach-driven self-oriented motives (“gain approval”) displayed similar correlations as avoidance-driven self-oriented motives (“avoid rejection”). The notion that these motives can be regarded as self-oriented is substantiated by the finding that they relate more strongly to self- than other-oriented unmitigated communion. Accordingly, controlling for self-oriented unmitigated communion (Study 2) lowered the correlations between items 1−4 and PA substantially (partial correlations: 0.17 < *r* < 0.22), but controlling for other-oriented unmitigated communion did not (partial correlations: 0.23 < *r* < 0.43).

**TABLE 6:** Correlations of Healthy Selfishness and Pathological Altruism scales with items for motivation and childhood antecedents across both studies.

		**Study 1**		**Study 2**			
**No.**	**Item**	**HS**	**PA**	**HS**	**PA**	**UC Self**	**UC Other**
	**Motivations for Helping Others**						
1	A major motivation why I give to others is to please them.	−0.17	0.46	−0.16	0.37	0.41	0.38
2	A major reason why I help people is to gain approval from them.	−0.28	0.56	−0.18	0.44	0.50	0.23
3	I often give to others to avoid criticism.	−0.34	0.62	−0.24	0.41	0.48	0.16
4	I often give to others to avoid rejection.	−0.36	0.63	−0.29	0.48	0.53	0.23
5	A main motivation why I give to others is to increase my openness to new experiences.	0.28	0.09	0.21	0.12	0.11	0.19
6	A main reason why I help others is a desire for personal growth.	0.30	−0.01	0.19	0.11	0.09	0.22
7	I like helping others because it genuinely makes me feel good to help others grow.	0.25	−0.01	0.18	0.09	0.09	0.39
	**Overly Nurturant Helping Behaviors**						
1	My helping sometimes causes others harm.	−0.12	0.47	−0.19	0.41	0.24	0.06
2	People often tell me to stop helping them, because they are overwhelmed with my constant helping.	0.07	0.48	−0.09	0.45	0.29	0.15
	**Childhood Antecedents**						
1	As a child, I was often encouraged by my family to substitute my own needs for their own.	−0.25	0.50	−0.19	0.36	0.31	0.18
2	As a child, I was often encouraged by my cultural environment to substitute my own needs for their own.	−0.25	0.51	−0.12	0.31	0.27	0.16

**N* = 891. Correlations greater *r* = 0.07, *r* = 0.09, and *r* = 0.11 are significant at *p* = 0.05, *p* = 0.01, and *p* = 0.001, respectively.*

Helping motivation items 5–7 are more strongly related to HS than PA across both studies. These items target growth and an intrinsic satisfaction from helping (e.g., “A main reason why I help others is a desire for personal growth,” “I like helping others because it genuinely makes me feel good to help others grow”) rather than receiving positive feedback or avoiding negative feedback from others. Also, they are more related to other- than self-related unmitigated communion, albeit the relationships with unmitigated communion are not as strong as those reported above.

Taken together, it can be tentatively concluded that helping others for instrumental, self-oriented reasons (gain approval, avoid rejection) is more related to PA, whereas helping others for intrinsic, other-oriented reasons (self-actualization and growth) is more related to HS.

This is also reflected in the correlations with two items assessing overly nurturant, potentially other-harming altruistic behavior^2^ (“My helping sometimes causes others harm,” “People often tell me to stop helping them, because they are overwhelmed with my constant helping”): while these items were unrelated or only weakly negatively related to HS, they were substantially *positively* associated with PA across both studies, and, to a lesser extent, also self-oriented (but not other-oriented) unmitigated communion. Taken together, this demonstrates that PA is not only associated with self-oriented motives for helping others, but is also associated with helping behaviors that can be harmful to others.

Finally, the two items on childhood antecedents show that substituting one’s own needs for those of others (family, cultural environment) is a powerful predictor of PA (and, to a lesser extent, self-oriented unmitigated communion) and at the same time negatively associated with HS. This, again, underpins the notion that PA is mainly driven by self-oriented motives, which might have been deprived early in people displaying high PA.

## Discussion

The main aim of the current investigation was to construct new measures to reliably and validly measure individual differences in two forms of paradoxical selfishness that have lacked measurement: healthy selfishness and pathological altruism. A secondary aim was to look at the different nomological networks of these two constructs. We predicted that measures of these constructs could be differentiated from related constructs in the field, and that each construct would be paradoxical in their correlations with other variables. In particular, we predicted that healthy selfishness would be related to higher levels of personal well-being as well as prosocial motivations for helping others, and that pathological altruism would be related to selfish motivations for helping others and maladaptive psychosocial outcomes as well as helping behaviors that tend to be harmful to others. We largely found support for our predictions.

Healthy selfishness was negatively related to unmitigated communion, suggesting that those with a healthy form selfishness tend to report less self-neglect and overconcern with the problem of others. Healthy selfishness had additional prediction value above and beyond unmitigated communion, however, particularly in its prediction of psychological well-being.

While healthy selfishness was related to a variety of indices of adaptive psychological adjustment—including life satisfaction, positive relationships, self-esteem, and authentic pride— it was independent from pathological selfishness (“I know I love rewards in life, even if there is a cost to others”; Raine and Uh, 2018, p. 513) and hubristic pride. Healthy selfishness was negatively related to vulnerable narcissism, and was only weakly positively correlated with grandiose narcissism and communal narcissism. From the perspective of the interpersonal circumplex, healthy selfishness was associated with a friendly assertive interpersonal style.

In line with our predictions, healthy selfishness was paradoxically related to greater prosociality across a number of dimensions. For one, healthy selfishness was significantly correlated with Big Five agreeableness and the Light Triad, a personality trait reflecting a loving and beneficent orientation toward others (Kaufman et al., 2019). Also, healthy selfishness was positively associated with growth-oriented and intrinsically enjoyable reasons for helping others, and negatively predicted self-oriented motivations for unmitigated communion.

This positive relationship between self-care and care for others is consistent with the work of Crocker and Canevello (2008, 2018). While they argue that the “compassionate goals” that are activated by the “ecosystem”— goals that are supportive, constructive, and do not harm others— are primarily about the genuine well-being of others, their research found that compassionate goals are significantly correlated with several facets of *self-compassion*, including mindfulness, self-kindness, and a sense of common humanity (Crocker and Canevello, 2008). Their research, along with the research we conducted, suggests that self-care and other-care may be strongly tied to the same overall system of care, an intriguing area for further research to explore.

As Crocker and Canevello (2018) note, “A key characteristic of goals in the ecosystem is that they are good for the self and others; any goal that requires people to sacrifice their own well-being for the sake of others is probably not motivated by the ecosystem, because it is not sustainable over time and is not good for the self and others” (p. 80).

In contrast, and as expected, pathological altruism was strongly positively correlated with unmitigated communion, particularly self-oriented reasons for unmitigated communion. However, pathological altruism displayed a much stronger prediction of pathological outcomes such as depression than unmitigated communion (unmitigated communion was more tied to fear within the normal range).

Also in line with our predictions, the underlying motivations of those scoring higher in pathological altruism reflected socially related fears rather than a willfully exploitative motivation. While pathological altruism was positively correlated with overly nurturant and potentially harmful helping behaviors, pathological altruism was independent of pathological selfishness. Nevertheless, in line with the old adage “the road to hell is paved with good intentions,” we found that pathological narcissism was significantly positively associated with the following items: “My helping sometimes causes others harm” and “People often tell me to stop helping them, because they are overwhelmed with my constant helping.”

Pathological altruism was particularly associated with aspects of *vulnerable narcissism*— including the need for admiration and a high frequency of shame— as well as fear of rejection, losing emotional contact, and losing control. These fears were reflected in the reasons for helping others: those scoring higher on pathological altruism were much more likely to report that they help others to avoid rejection and criticism and to gain approval and to please others. This was also reflected in the moderate correlation between pathological altruism and the self-sacrificing self-enhancement facet of the Pathological Narcissism Inventory (Pincus et al., 2009), and with the positive (although weaker) correlation with communal narcissism (Gebauer et al., 2012). Developmentally speaking, we found tentative evidence to suggest that these adult motivations may be rooted in the early childhood experiences of constantly being asked by one’s family and the overall culture to substitute one’s own needs for the needs of others, consistent with predictions made by prior researchers (Kohut, 1971; Bachner-Melman and Oakley, 2016).

Pathological altruism was less clearly associated with the interpersonal circumplex compared to healthy selfishness. This finding parallels prior findings showing that unmitigated communion is not as neatly placed on the interpersonal circumplex as unmitigated agency (Helgeson and Fritz, 1999). Helgeson and Fritz (1999) suggest that it may be difficult to locate unmitigated communion on the interpersonal circumplex “because unmitigated communion is theoretically associated with a lack of personal agency (i.e., focus on the self) rather than a lack of *interpersonal agency*, which is what the interpersonal circle measures” (p. 155). The same reasoning may apply to pathological altruism. Indeed, we found that pathological altruism was uncorrelated with the introversion-extraversion dimension of personality.

Alternatively, our findings may simply point to the conclusion that pathological altruism is more paradoxical than healthy selfishness, being tied to motives that may work at odds with each other, such as a drive to help others along with selfish motivations. In this sense, those with higher levels of pathological altruism may experience more inner conflict than those with healthy selfishness, but that is an issue for further investigation.

Also notably, we did not find any significant sex differences in either healthy selfishness or pathological altruism. This is interesting considering that prior research has found women tend to be more altruistic than men in dictator games and on self-report questionnaires, whereas men tend to be more selfish than women on these measures (Rand et al., 2016; Brañas-Garza et al., 2018; Kaufman et al., 2019; but see Balliet et al., 2011). Our findings may just be another indication of the distinction between altruism and *pathological* altruism on the one hand and selfishness and *healthy* selfishness on the other hand. It is possible that sex differences are much more minimal, if nonexistent, on these more paradoxical forms of selfishness. This could be a promising line of further research.

## Limitations and Future Directions

This study has some limitations. For one, we relied exclusively on self-report measures. Future studies should also assess other-reports and behavioral correlates, such as in interpersonal interactions, of both constructs, and look at the stability of these traits over time. What’s more, future studies should include more external measures of clinical outcomes (e.g., actual history of diagnosis) as well as indicators of well-being, resiliency, and life experiences. We only took a very tentative look at some potential developmental experiences that might underlie these paradoxical forms of selfishness, but future research would benefit from looking at a wider array of developmental experiences that have an impact.

Also, while both constructs were moderately negatively correlated with each other, there is still plenty of room for one’s mix of healthy selfishness and pathological altruism to vary at a within-person level of analysis. It might be fruitful to look at whether different clusters or latent classes predict important variables of interest above and beyond simply looking at the between-person level of analysis.

Regarding the associations between PA and the interpersonal circumplex scales reported here, one might have expected a stronger association with the communion-axis of the circumplex. However, upon closer consideration, such associations might emerge with interpersonal *problems* (Horowitz et al., 1988) rather than interpersonal behavior *per se* (as also reflected in the items measuring overly nurturant behavior in this study), which could be included in future studies. Also, the significant link between pathological altruism and both neuroticism and vulnerable narcissism (Miller et al., 2017) would not be reflected in the interpersonal circumplex.

Another potential limitation of the current study may be the wording of the items of the scales. We devised items on the basis of conceptual considerations, using common phrases from natural language. These depict prototypically high expressions of HS and PA. While an advantage of this naturalistic approach is that HS and PA are readily relatable to one’s own experience and behavior, a disadvantage may be that low expressions are not unambiguously defined in each case. For instance, while the high pole of PA item No. 6 “I have little time to myself because I am too busy helping everyone” is clearly related to PA, the low pole is not unambiguously related to low PA.

To check for potential nomological network validity differences along the trait dimensions, we conducted complementary median-split correlation analyses for the variables listed in Table 4. The validity profiles between high and low trait expressions of HS and PA were generally very similar. Media-split HS profiles correlated at *r* = 0.81, PA profiles *r* = 0.94. Thus, it can be tentatively concluded that nomological networks are highly similar between high and low trait expressions. However, future studies could directly address the question of content validity using qualitative techniques such as think-aloud protocols while participants are thinking through their responses to the items.

Finally, the concepts and scales presented here have arisen in a western cultural context, which puts stronger weight on independent than interdependent orientation (Singelis, 1994). Not all of the findings reported here might thus generalize to more interdependent contexts, which might be particularly true for the associations between overly altruistic behavior and indicators of psychological maladjustment.

## Clinical Implications

The strong association of pathological altruism to depression and social fears suggests there may be clinical implications of these constructs. Recent research has pointed out the clinical implications of narcissism, particularly vulnerable narcissism (Jauk and Kaufman, 2018; Kaufman et al., 2018). Since we found a strong correlation between pathological altruism and vulnerable narcissism, the same recommendations that apply for vulnerable narcissism may also apply here, such as helping those scoring high in pathological altruism have a more stable self-esteem, increase healthy assertiveness of one’s own needs, and increase psychological flexibility (the opposite of experiential avoidance; see Hayes et al., 1999).

Our results suggest an additional path by which those with high levels of pathological altruism may not only decrease their levels of depression and fear but also increase their well-being: *increasing their levels of healthy selfishness*. Helping those who suffer from high levels of pathological altruism learn that it’s health and even growth-fostering to take care of oneself and enjoy life’s little pleasures may go a long way in helping these individuals feel less shame when thinking about themselves and their own needs. We think this could be a promising avenue for future research.

## Conclusion

Here, we presented new scales for two understudied forms of selfishness: healthy selfishness and pathological altruism. The scales display good reliability and validity with respect to related constructs. Importantly, validity analyses underpin the paradoxical nature of both constructs as they show that not all selfishness is necessarily bad, and not all altruism is necessarily good. Healthy selfishness is largely associated with indicators of adaptive psychological functioning and genuine prosocial orientation, whereas pathological altruism is associated with maladaptive functioning, vulnerable narcissism, and helping behaviors that might be harmful to one’s self and to others.

## Data Availability Statement

The datasets generated for this study are available on request to the corresponding author.

## Ethics Statement

The studies involving human participants were reviewed and approved by the University of Pennsylvania’s institutional review board. The participants provided their written informed consent to participate in this study.

## Author Contributions

SK conceived of the study idea, designed the experiment, set up the study, and ran the study. SK and EJ contributed to the generation of hypotheses, the collation of the results, the data analysis, and the writing of the manuscript.

## Conflict of Interest

The authors declare that the research was conducted in the absence of any commercial or financial relationships that could be construed as a potential conflict of interest.

## Footnotes

1 We note that the CFI is rather low, which is likely to the high number of indicators. Nevertheless, we refrained from reducing the number of indicators (by means of parceling, for instance) as we wanted to evaluate the factor structure for the full set of items which were selected on the basis of exploratory factor analyses in Study 1.

2 These two items were originally part of the initial PA item pool, but were not included in the scale as they not only had high item difficulty, but also go beyond the proposed construct definition. Nonetheless, we consider these items important validity indicators for the PA scale.
